# Nanocrystal-induced chronic tubular-nephropathy in tropical countries: diagnosis, mitigation, and eradication

**DOI:** 10.1186/s40001-023-01162-y

**Published:** 2023-07-05

**Authors:** Sunil J. Wimalawansa, Chandra B. Dissanayake

**Affiliations:** 1 Cardiometabolic & Endocrine Institute, Department of Medicine, Endocrinology, Nutrition, (formerly UMDNJ/Rutgers University), New Brunswick, NJ 08902 USA; 2 Present Address: Moratuwa, Sri Lanka; 3 Department of Geology, University of Peradeniya, Peradeniya, Sri Lanka; 4 Institute of Fundamental Studies, Kandy, Sri Lanka

**Keywords:** Chronic renal failure, CKD, Environment, Fluoride, Geochemistry, Hard water, Nephropathy, Tropical countries, Tubulointerstitial

## Abstract

In tropical countries, a mysterious tubulo-interstitial chronic renal disease (CKD), unrelated to diabetes, hypertension, and immunological causes, manifested four decades ago. Approximately 25,000 primarily middle-aged male farmers succumb annually to this crystal-tubular nephropathy (CTN). Without any known causative factors, it was identified as CKD of unknown aetiology (CKDu). Because multiple factors contribute to causing it later, was changed to CKD of multi-factorial (CKDmfo). Despite no evidence, it was hypothesised to cause by agrochemicals or heavy metals in food or drinking contaminated water. However, current data suggest that the CKD-CTN is due to natural geogenic water contamination. Consumption of concentrated stagnant groundwater from deep-dug wells and tube wells containing hard water and fluoride, overdecades is necessary for its clinical manifestations. In all affected countries have prolonged annual dry seasons that led to the evopo-concentration of ions and minerals in groundwater, making hard water even more unpalatable, thus, peasants consume lesser amounts of water. They develop chronic dehydration from daily exposure to hot climatic conditions aggravated by regular alcohol intake. These conditions provide a highly conducive environment—a perfect storm for calcium phosphate (CaPO_4_) crystal formation in renal tissues. Our recent histological and preliminary electron microscopic data reveal deposition of CaPO_4_ crystals and nano-tubes in kidneys. While CaPO_4_ nano-minerals are unstable, the presence of fluoride ions stabilises and allows their growth. This new concept paves the path for highly cost-effective, straightforward local solutions to protect farm workers and eliminate the disease, without embarking on expensive medications, interventions, or building hospitals. Chronic dehydration-associated CKD–CTN is preventable by increased consumption of potable water. Increasing clean water consumption reduces CKD–CTN incidence, and associated morbidities and premature deaths. However, the damage becomes irreversible when the disease advances beyond CKD stage IIIB. The incidence of this deadly renal failure can be prevented by its education, lifestyle changes, and increased water consumption, not by treating the renal disease or expanding dialysis centres/hospitals, or transplantation services. Eradication of CKD-CTN cost significantly less than the current approach of treating affected persons and unnecessarily expanding health infrastructure. Since the manifestation of CKD-CTN is due to consuming naturally contaminated drinking water (with calcium containing hard water and fluoride), it is not difficult to remove these to prevent CKD-CTN: thus, international assistance is unwarranted for its eradication. The straightforward approaches described here will prevent CKD–CTN and save thousands of lives in affected farming communities.

## Keypoints

- During the past four decades, an unusual tubulointerstitial chronic renal disease (CKD) unrelated to diabetes, hypertension, or immunological causes, manifested in over twenty tropical countries located north of the equator.
- Previously, this crystal-tubular nephropathy (CKD–CTN) was assumed to arise from agrochemical or heavy metal contamination.
- Natural geogenic conditions/events contaminate groundwater. Drinking such concentrated, stagnant groundwater in the presence of chronic dehydration over a prolonged period leads to the development of CKD–CTN.
- Nano-minerals stabilised with fluoride ions grow perniciously in kidney tissues in the presence of dehydration. It provides a conducive environment for CaPO_4_ crystal formation in renal tubules and interstitial tissues.
- In conjunction with lifestyle changes and providing affordable clean drinking water will prevent the disease and eventually eradicate CKD–CTN. Those with CKD stages below stages IIIB can reverse by drinking plenty of clean water. These approaches would save thousands of lives in affected communities and healthcare costs.

## Introduction

Over forty million people in tropical countries who consume groundwater naturally contaminated with geogenic components are at risk of developing an unusual form of chronic kidney disease (CKD). Most affected are adult males in farming communities in affected tropical countries. They create unique chronic tubulointerstitial chronic kidney disease (CKD–CTN) unrelated to commonly known factors, such as diabetes, hypertension, toxins, or immunological disorder. Each year approximately 25,000, primarily middle-aged males, die from it [1].

However, it neither exclusively affects agricultural communities nor is it related to agrochemicals. Those who were never involved with agriculture and engaged in other professions also developed this fatal CKD; what matters are their location and drinking water source—the quality of water, and behaviour [2]. There is no affirmative and reliable scientific evidence that fertilisers, arsenic, pesticides, arsenic, heavy metals, algae toxins, genetic susceptibility, ayurvedic or traditional medications, other known chemical nephrotoxins, or direct human interventions cause CKD–CTN [3].

**Fig. 1:**
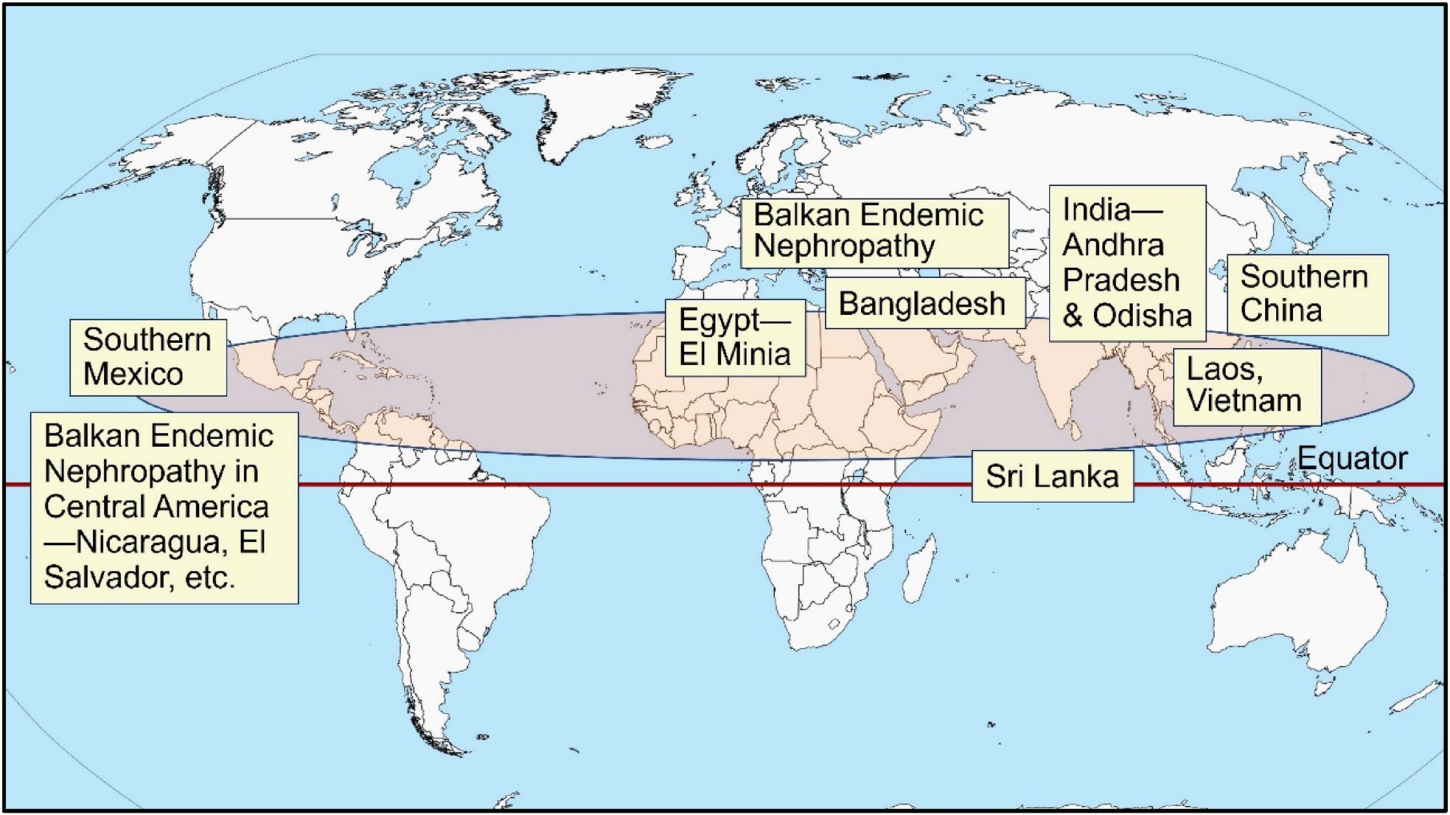
Schematic illustration of the geographical distribution of CKD–CTN-affected countries. More than 90% of these are located north of the equator, in proximity to it

There had been over thirty conjectures proposed as the cause for CKD–CTN; none were examined meticulously or proven to be the cause. Since the exact cause was unknown, it was initially identified as CKD of unknown etiology (CKDu) [4, 5]. Subsequent data firmly pointed towards the need for multiple factors to precipitate CKDu; hence, it was re-named CKD of multi-factorial etiology (CKDmfo) [1, 6, 7]. This fatal renal failure primarily affects countries that are geographically restricted—located north and closer to the equator (Fig. 1).

The similarities of diseases and characteristic differences between the central (meso)American and Sri Lankan nephropathy have been described [8, 9]. Most recent data confirmed that prolonged exposure to multiple factors (usually more than 10 years), including environmental conditions, chronic dehydration, and favorable internal milieu is necessary to develop CKD–CTN [10]. These various factors lead to the precipitation of CaPO_4_ crystals and nano-tubes in renal tubules and interstitial spaces in renal tissue, causing chronic renal failure [10].

Ongoing research suggests that it is a geo-environmental-induced water-borne disease. CKD–CTN is due to inflammatory CaPO_4_ nano-particles in renal tubular tissues [10, 11]. For mineral precipitation, it is necessary to have prolonged dehydration and low urine output. Urine pH of around 6.8, hypercalcemia, hypercalciuria, hypomagnesuria, and low urinary Mg^2+^-to-Ca^2+^ ratio, these conditions are favourable for CaPO_4_ precipitation in the kidneys [12].

Crystal induces chronic inflammation and oxidative stress, inflammatory cell infiltration and renal tubular cell damage, apoptosis, and tissue fibrosis; causing renal shrinkage, leading to chronic renal failure (CRF). With the advancing knowledge and considering the above, we coined the terminology CKD–crystal tubular-nephropathy (CKD–CTN) to describe this disease. This acronym parallels CKD–mineral and bone disorders (CKD–MBD) [13] and is logical [14]. Whereas, when there is no scientific evidence or correlation between agrochemicals (or heavy metals) and CKD–CTN, attempted usage of agriculture-based acronyms, such as CINAC, ACN, KDUCAL, NUCAL, etc., is flawed, inappropriate, and misleading [3, 15–17]. Such diversion does not help peasants but further stigmatises farming communities. Even abbreviations CKDu and CKDmfo are superfluous as these do not describe an etiology.

## The cause of CKD–CTN

### The role of Ca^2+^, PO_4_^3-^ and F^−^ in nano-crystal formation in the kidney

In the affected communities, consumed groundwater has high dissolved solids and electrical conductivity, and Ca^2+^, phosphate (PO_4_^3^), and fluoride (F^−^). For example, the median range of these in groundwater, in affected regions are TDS 500 mg/L, EC 1,800 μS/cm, calcium 125 mg/L, Mg^2+^ 50 mg/L, alkalinity 250 mg/L, SO_4_^2-^ 200 mg/L, F^−^ 0.8 mg/L, and PO_4_^3-^ 0.025 mg/L [18]. Most of these water sources are unhealthy to consume over time, and mentioned components are above the EPA and WHO-mandated safety standards [18]. However, no reliable published research studies reported higher (or even detectable) levels of agrochemicals, heavy metals, or other known nephrotoxins [3, 19–23]. Besides, because of the unpalatability of water, people in these hot tropical climatic regions consume less water, and the habit of daily alcohol consumption worsens dehydration [11, 24].

Under the conditions mentioned above, renal tissues chronically exposed to high concentrations of Ca^2+^, (PO_4_)^3-^, and F^−^ provide a conducive milieu for calcium phosphate (CaPO_4_) and other nano-mineral crystal formation. Although CaPO_4_-hydroxyapatite formation in kidneys is not unusual: these structures are unstable [Ca_5_(PO_4_)_3_(OH) = Ca^2+^  +  3(PO_4_)^3−^  +  OH^−^] under the normal range of urine pH. In these regions, groundwater contains higher F^−^ concentrations [20, 25] but is sporadic [8, 9]. At the proper concentrations, F^−^ substitute hydroxyl or PO_4_ [3] groups in CaPO_4_ hydroxyapatite through ionic and hydrogen bonds form more stable fluorapatite crystals [fluoroapatite: 5Ca^2+^  +  3(PO_4_)^3−^ +  Ca_5_(PO_4_)_3-_F] [10].

This phenomenon is analogous to incorporating F^−^ into CaPO_4_ apatite in the enamel component in teeth and skeletal tissues [8, 9]. It strengthens existing hydroxyapatite scaffolds despite requiring a minute F concentration (e.g., between one and three mg/L). It produces stable fluoroapatite crystals resistant to decay [8, 9], and known to grow gradually. Consequent cell-mediated fluorapatite, carbonatoapatite and incorporation of matrix proteins make these nano-crystals stiffer [26]. In teeth, incorporating F^−^ leads to brownish discolouration of teeth and bones to become brittle [8, 9, 27].

### Nano-crystal and nano-tube formation in renal tissues

**Fig. 2:**
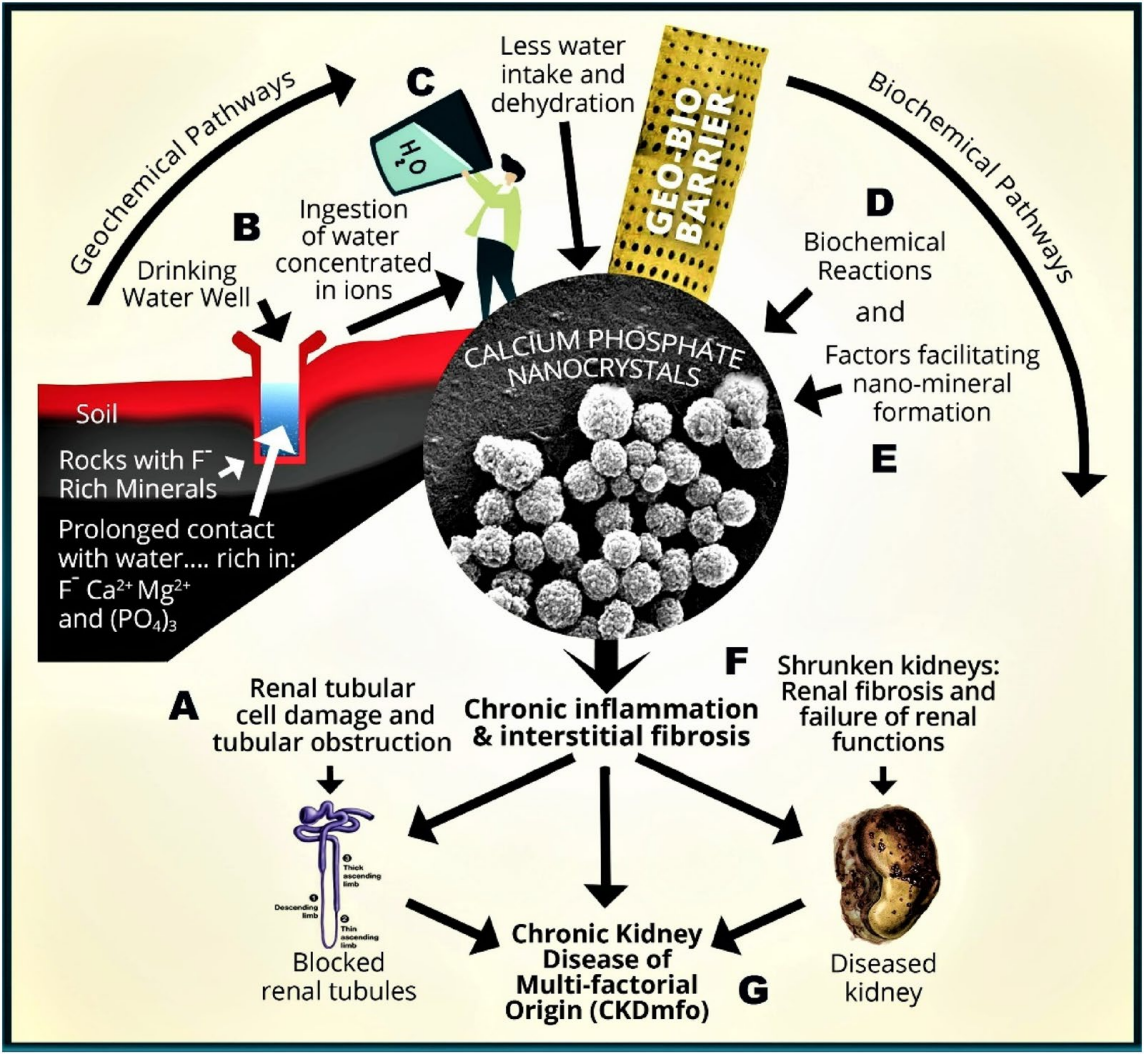
Illustrates the geological and biological (Geo–Bio) pathways of forming nano-tubes and nano-crystals in CKD–CTN. A conceptual diagram is illustrating ways for the formation of calcium phosphate (CaPO_4_) nano-crystal-based hydroxyapatite in renal tubules causing CKD–CTN: **a** interaction of rocks containing minerals rich in F^−^, Ca^2+^, Mg^2+^, PO_4_^3^^−^ with groundwater; long duration of water–rock interaction is exacerbate by seismic effects; **c** consumption of water with high concentrations of ions; **d** biochemical reactions and super-saturation, precipitating CaPO_4_ nano-crystals and nano-tubes in kidney tubules; **e** common factors enhancing the nano-mineral precipitation; **f** Low-grade persisting chronic inflammation and tissue fibrosis; and **g** Onset of chronic renal failure (adapted from Wimalawansa and Dissanayake, 2022; Frontiers of Water and human health) [10]

Ultra-structural studies in human kidney tissues with end-stage CKD consistently reported 50 to 1500 nm multi-lamellar mineral particles [28]. Presence of CaPO_4_ crystals was also reported in renal biopsy samples in those with several chronic renal diseases [14, 29–31]. While the generic crystal formation in renal tissues is not unique, the condition leading to crystal formation and classic pattern of CRF in CKD–CTN are unique [10]. Mentioned nano-mineral particles of polycrystalline CaPO_4_ are produced in both extracellular matrices and tubular cells [10, 30, 31]. Figure 2 shows a conceptual diagram illustrating Geo–Bio interactions, pathways, and conditions necessary to precipitate CaPO_4_ nano-crystal in renal tissues in renal tubules, causing CKD–CTN. These are not present in other types of nano crystallisation of minerals in the kidney.

Hydroxy- and fluorapatite nano-particle growth occurs slowly in vivo. Generally, it takes years before producing symptoms [32–34]. These are inflammatogenic and, thus, attract fibroblasts and leukocytes, low-grade inflammation, tissue fibrosis, and eventually renal failure. In vivo*,* nano-crystal complexes attract matrix proteins [35] incorporated into hydroxy-fluorapatite, further protecting nano-crystal dissolution and from enzymatic degradation [36, 37]. These allow a steady growth of fluoroapatite crystals, eventually blocking and perforating renal tubules, causing intense local inflammation, cellular apoptosis, and fibrosis [10].

### Diagnosis of CKD–CTN

The diagnosis of tubule-interstitial nephritis/nephropathies (TIN) is generally considered when there are no common causes present in the history and presented with unexplained renal failure dysfunction [38–40]. Routinely used urine and serum biochemical makers, such as urine and renal profiles capture all types of CKDs, but none are specific to CKD–CTN. Similarly, none of the routinely used in vivo imaging and scanning methods, distinguish between CRF.

A history of exposure, living location and clusters of persons with CRF, and the environmental circumstance should lead to the clinical suspicion of CKD–CTN [41]. History of not having diabetes and hypertension, lack of history of exposure to known nephrotoxins (most are acute onset), snake bites and immunological disorders virtually exclude most common types of CKDs. The primary causes of TIN are illustrated in Table 1.

**Table 1:** Primary causes of tubulo-interstitial nephritis (TIN)

Causes	Examples
Infections	Bacterial pyelonephritis; Hantavirus; Leptospirosis; Tuberculosis
Drug-induced	Analgesics; lithium; Cyclosporine: certain Chinese herbs; Chemotheraputic agents; Toxins; Heavy metal (e.g., Lead, Cadmium)
Immune-mediated	Sjogren’s syndrome; Anti-tubular basement membrane disease
Metabolic disorders	Gout; Hypercalaemic interstitial nephritis; Hypokalemic interstitial nephritis
Lifestyle issues	Chronic dehydration, daily alcohol intake
Hereditary disorders	Wilson’s disease; Hyperoxaluria; Cystinosis
Hematologic disorders	Amyloidosis; Sickle cell disease; Light chain nephropathy; Cast nephropathy
Miscellaneous	Crystalline tubular-nephropathies: Mesoamerican nephropathy; Balkan nephropathy—CKDu/CKDmfo

Proteinuria is an early manifestation of glomerular diseases, such as diabetes and hypertension, but protein in urine appears late in renal tubular disorders [8, 41–43]. Consequently, using proteinuria of 30 mg/L as a cutoff point, which is standard for glomerular renal disease [44, 45], is inappropriate for CKD–CTN [8, 46]. Using such would significantly underestimate the diagnosis and prevalence of CKD–CTN in the community [26], as proteinuria is a later finding of CKD-CTN. This significantly delays the diagnosis of CKD-CTN, which is detrimental for the prognosis. Standard diagnostic method even with renal biopsies with routine histology would not differentiate CKD–CTN from other CKDs; as crystals are not visualised under standard microscopy and magnifications.

The characteristic interstitial infiltrate comprises lymphocytes, macrophages, eosinophils, and plasma cells, which can rapidly transform into interstitial fibrosis leading to chronic kidney disease (CKD). The critical biochemical differential diagnosis aid is tubular-specific markers in urine, which is straightforward and not expensive [9, 41, 47]. Examples of tubular specific makers (when the primary damage is to renal tubules) include Cystatin C, Kidney injury molecule-1 (KIM-1), Monocyte chemotactic protein-1 (MCP-1) N-acetyl-β-D-glucosaminidase (NAG) Nephrin, Netrin-1, Neutrophil gelatinase-associated lipocalin (NGAL) Smooth Muscle alpha-Actin (α-SMA), Urinary Vitamin D binding protein (uVDBP), and Vascular endothelial growth factor (VEGF) [41, 48, 49].

### Mechanisms of formation of nano-tubes in renal tubules

In 2019 the authors postulated that repeated chronic dehydration generated peaks of Ca^2+^ within renal interstitial tissues and in tubules and excess PO_4_^−3^, intensifying the formation of nano-crystals and nano-tube formation and growth. This established the inflammatory process attracts fibroblasts and tissue fibrosis, causing CKD–CTN and premature deaths [10]. This process requires over a decade of consuming contaminated water containing higher mineral content. 

The formation of intra-tubular Ca^2−^ and other minerals increases the risks for clinically significant mineral crystallisation in kidneys. A charitable foundation headed by the lead author, Water Board, and others have provided potable water to CKD–CTN-affected villages through reverse osmosis units. These efforts have led to over a 50% reduction in two to three years: the improvement was continued [50].

With higher intakes of mentioned minerals, PO_4_^-3^ and F^-^, at the body temperature the solubility of the “Ca^2+^ PO_4_^3−^” product exceeds a threshold thus, leads to the formation of CaPO_4_ nano-particles. In addition, super-saturation of CaOx, urates, or other minerals also occurs, albeit at a lesser quantities than CaPO_4_ -hydroxy apatite in renal tissues. When nano-crystals exceed 1 mm, such accumulations can be visualised with in vivo imaging methods, such as high-resolution computed tomography [14, 29, 51]: Nano-particles, even when combined, are too small to be visible in other routine radiological investigations. It takes over a decade to develop symptomatic renal disease and full-blown renal failure [22, 52]. However, renal biopsy with high-resolution computed tomography or electron microscopic studies can confirm CKD-CTN diagnosis, early in the disease.

### Magnesium deficiency increases the risks of renal nano-crystal formation

Mg^2+^ competes with Ca^2+^ in physiological situations and is a cofactor for enzymatic reactions: it is also essential for releasing hormones from endocrine cells. Under the above-discussed conditions, hypomagnesemia enhances the accumulation and precipitation of Ca^2+^ within renal cells. Moreover, with saturation in stagnant tissue fluids within the counter-current multiplier mechanisms in kidneys, the lack of antagonist effect of Mg^2+^ on Ca^2+^ synergises CaPO_4_ nano-crystal formation in renal parenchyma.

When rats are fed an Mg-deficient diet, the harmful effects of calcium are accentuated and worsened by low dietary potassium. Likewise, circulatory Mg^2+^ concentration is reduced when rats are fed high-calcium diets [53]. In contrast, renal accumulation of calcium (CaPO_4_ and other minerals) is mitigated with Mg^2+^ supplements. Contrary to suggestions based on incidental and isolated findings [23] and an animal study [54, 55], Mg^2+^ does not cause CKD–CTN. Instead, magnesium protects renal tissues from excess Ca^2+^ [56–58] and against mineral crystallisation [10]. Also, physiological intra-cellular Mg^2+^ concentration is essential for proper biological and physiological activities in all cells [59–61].

Besides, lower circulatory total magnesium Mg^2+^ concentrations usually measured in blood neither correlate with the intracellular Mg^2+^ nor its biological activity [60, 61] and facilitate CaPO_4_ precipitation. In addition to dietary factors, these data emphasise the importance of the quality of drinking water, multiple ion interactions, and their ratios and competition in vivo physiological situations—multi-factor effects. These either mitigate or aggravate the ionic reactions, nano crystallisation, and clinical outcomes [10, 59, 61].

### Dietetic fructose in renal tubular CaPO_4_ nanotube formation

Rats fed on an Mg-deficient diet develop hypomagnesemia, and clinical outcomes are worsened when fed with fructose (to a lesser extent with glucose) than those fed unrefined, complex carbohydrates. In the fructose-fed group, renal calcium content was eight times greater than in the control group [62]. High fructose diet also worsens existing hypercalcemia, hypercalciuria, and hypomagnesuria, enhancing CaPO_4_ crystallisation in kidneys and renal failure. Excess fructose in the presence of low Mg^2+^ increases risks for nephrocalcinosis and renal failure [59, 62–64].

Meanwhile, higher F concentrations increase Ca^2+^ precipitation in soft tissues, arteries, and kidneys. Those who live in regions with a higher prevalence of CKD–CTN are economically poor, and their diets are predominantly carbohydrates. In addition, these diets are deficient in micronutrients and Mg^2^, whereas fructose content is high and represents a significant component of their carbohydrate intake. In the presence of low cell/tissue Mg^2+^ concentration, higher fructokinase activity further increases the risks of renal calcification [11].

### Synergistic ion interactions exacerbate the nano-crystal formation

Physiological concentrations of Mg^2+^ counteract the adverse effects of increased intracellular Ca^2+^ concentration and modulation of Ca^2+^ channels. Hypomagnesemia increases N-methyl-D-aspartate receptors and nuclear factor-kappaB activity, stimulating the renin–angiotensin system and reducing renal blood flow [64]. Aggregated apatite nano-tubes attach to the luminal cell surfaces [30], causing blockage and/or rupturing renal tubules and reducing renal functions.

Insufficiency of crystallisation modulators, such as Tamm–Horsfall protein, osteopontin, sodium phosphate co-transporter, or sodium–hydrogen exchanger regulatory factor-1, also increases the risks of tubular and interstitial nephrocalcinosis [65]. Whereas the crystal-induced interstitial tissue inflammation and oxidative stress further reduce intra-renal blood flow via activation of the renin–angiotensin system, causing further impairment of renal functions.

### Other contributory factors

Renal failure increases PO_4_ retention, which leads to increased Klotho levels. Meanwhile, advanced renal failure disrupts fibroblast growth factor-23 (FGF23), signalling that increases the accumulation of PO_4_ [66]. Therefore, modulation of Klotho activity should be investigated as a target for intervention in those with moderate CKD–CTN. For example, using Klotho or its synthetic agonist reduces PO_4_ toxicity and severity in CKD–CTN and accelerated ageing [67].

**Fig. 3:**
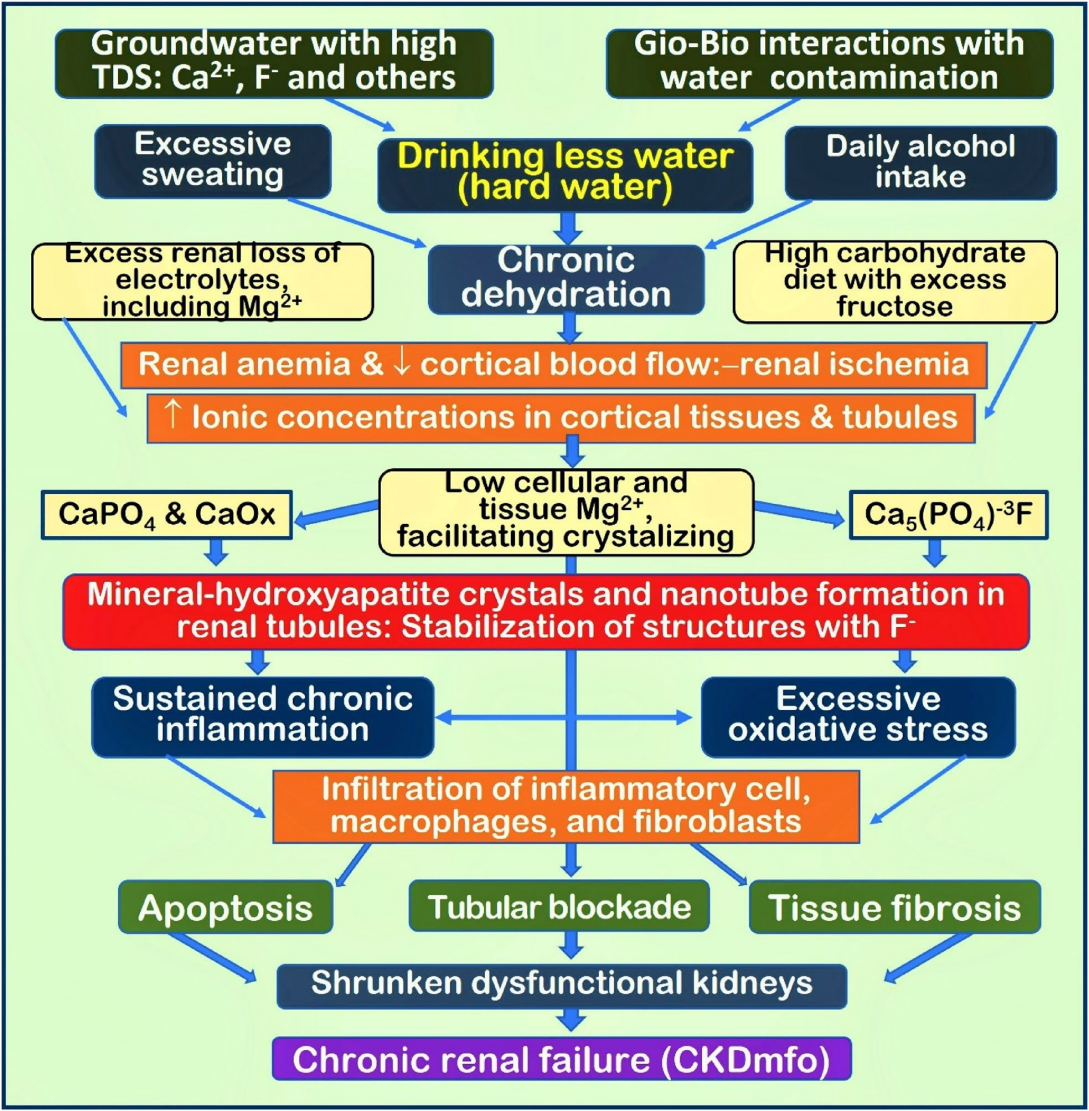
Multiple pathways and interactions leading to nano-mineral forming in renal tubular and cortical tissues. Chronic dehydration-induced high mineral concentration within the renal fluid and tissues is conducive to forming mineral crystals. Multiple factors aggravate renal ischemia, anaemia, and inflammatory cell infiltration. These leads to chronic inflammation and oxidative stress. Collectively, these lead to disruption of tubular cell functions and fibrosis, causing CKD–CTN [TDS: total dissolved solids; Ca^2+^ and PO_4_^3-^, Mg^2+^, etc.); CaPO_4_: calcium phosphate; CaOx: calcium oxalate; Mg^2+^: magnesium; F^−^: fluoride] (modified from Wimalawansa and Dissanayake) [10]

Hypomagnesemia worsens the retention of Klotho-induced PO_4_ tubular load and renal functions. Therefore, prophylactically correcting Mg^2+^ deficiency could be a cost-effective approach for those living in endemic areas to reduce PO_4_ load and an economical way to prevent and reverse CKD–CTN in its early stages. Figure 3 illustrates the fundamental mechanisms and pathways, forming CaPO_4_ crystals in renal tissue.

It is not climate change but the associated disasters that arose from the willful destruction of the environment through uncontrolled development and agriculture and irresponsible behaviour of people that endanger nature, causing many preventable human diseases. Since CKD–CTN originates from an environmental cause—natural Geo–Bio interactions (Fig. 2)—it is feasible to reduce the risk of CKD-CTN by addressing the root causes: without embarking on expensive pharmaceutical agents. As we and others have previously reported [1, 34, 68].

The most critical intervention is supplying potable water to all affected regions [8, 52]. This can be achieved via mentioned cost–benefit analysis and practical steps to help eradicate CKD–CTN [52, 69] and adopting previously described economic longer term chronic disease management programs [69, 70]. The following section describes ways to control and eradicate CKD–CTN.

### Eradication of CKD–CTN is straightforward

Interventions for CKD–CTN in affected regions need to focus on providing potable water at an affordable price and educating them on ways to avoid chronic dehydration. Providing centrally purified pipe-borne water to the affected areas is expensive and estimated to take more than three decades. Even then, the methodologies used by Water Boards unlikely to remove sufficient hardness (CaPO_4_) and fluoride from water. In the interim, the most cost-effective and practical way to provide a scalable potable water supply is through ionexchange systems, reverse osmosis or water descaling/softening to remove hardness, which can also eliminate excess F^-^ scattered across affected regions [46, 71, 72]. A program to educate the public, starting from schools and extending to the community, on avoiding harmful behaviour-like daily alcohol intake is critical to preventing the younger generation from getting affected.

In addition, when a breadwinner or any family member acquires CKD–CTN, their expenses escalate, and their income dwindles. This initiates a vicious cycle within the family that affects children’s education, and ability to do manual activities to generate revenue. Consequently, it escalates the poverty, escalating malnutrition: a universal phenomenon observed in all affected regions [73, 74]. Therefore, programs to alleviate CKD–CTN must encompass poverty alleviation. The authors recommend implementing straightforward, cost-effective muti-prong programs to prevent the fatal CKD–CTN [8, 10, 52, 74], as we previously described [52]. A broader holistic and affirmative approach based on the concepts described [46, 52], encompassing education, awareness, prevention of environmental pollution, lessening malnutrition, correcting unhealthy behaviours and habits that acquired during the past four decades, and providing clean water would be sufficient to reduce the incidence and eradicating this deadly disease rapidly from affected communities [75–77].

## The way forward and future research

Based on the aetiology as described here, future studies should focus on minimising the risks of mineral crystal formation in kidneys in affected regions. Such processes would lay a firm path and optimal ways to minimise human costs through public education, alleviate micronutrient malnutrition, and provide affordable clean drinking water to affected people. Such actions are a thousand-fold more economical than the current approach of waiting for people to develop and then treat the disease. It reduces the incidence of CKD–CTN in affected countries, as we previously described with feasibility and cost–benefit analyses[52].

Additive and synergistic effects and ion interactions, intertwined with multiple factors, trigger the onset of renal failure (Fig. 3). Therefore, in vivo models of CKD–CTN detailed biochemical studies that simulate the environmental and Gio–Bio conditions, using “bioavailable” components (not the “total” measured amounts), are necessary [7]. Noteworthy that in vitro chemical interactions are not necessarily extrapolatable to in vivo complex biochemical interactions and pathophysiology.

Additional in vitro biochemical and in vivo electron microscopic studies using renal tissues and bioavailable Ca^2+^, PO^−4^, and F^−^, simulating the CKD–CTN situations, are needed to assess potential antagonistic, additive, or synergistic interactions in the formation of nano-minerals to understand the exact mechanisms. Such would also reveal additional ways to eliminate this deadly condition.

### Some in vitro data and in vivo animal studies cannot extrapolate to humans

Compared to in vitro observations and reactions, dozens of compounds interact simultaneously in vivo to buffer, neutralise, and scavenge to function as agonists or antagonists. Numerous published examples have reported the inappropriateness of direct extrapolation of in vitro studies in isolation or in vivo small animal studies [1, 55], to in vivo human situations [70]; such interpretations could be misleading. Moreover, generalising such data is unreliable and insufficient for understanding complex multi-functional interactions in vivo to deduce clinically meaningful conclusions in humans.

Therefore, in vitro*,* laboratory examination of compounds in isolation is reasonable initially to generate hypotheses but should not be extrapolated to in vivo complex situations, such as CKD–CTN. This is analogous to interpreting data from individually isolated ayurvedic medications: such experiments would not generate meaningful and extrapolatable inferences. An example of CKD–CTN is mis-interpreting marginally higher concentrations of Mg^2+^ reported in sporadic drinking water samples [1, 20, 78] and data from non-reproducible laboratory rat studies [55]. Broader interpretation and generalisation of data from such experiments are not only unscientific and ambiguous but also unphysiological to make a sweeping conclusion that Mg^2+^ or F^−^ interactions alone cause CKD–CTN [8–10]. We provided proper experimental formats, capital and annual cost estimations, and written protocols to prevent CKD-CTN to successive three presidents in Sri Lanka, department of health and university staff. However, hardly any of these were implemented to date.

### Types of in vivo experiments needed now

Despite the established biochemical paths and physiological functions, there are different views of the role of Mg^2+^ on kidneys and the causation of CKD–CTN. Few consider higher Mg^2+^ in drinking water with or without F^-^ to cause CKD [54]. In contrast, most evidence supports that Mg^2+^ has a protective effect on kidneys [79–81]. To understand the accurate picture, researchers need to take into account ionic interactions, their ratios, and combinations (based on Gibbs free energy calculations) coupled with bioavailable (effective) components in (e.g., F^-^) in vivo models.

Data obtained using bioavailable components and the ratios of ions (e.g., Ca^2+^ to Mg^2+^) would allow a better understanding of in vivo biological interactions to improve clinical outcomes. These may explain the outcome discrepancies, such as renal damage with low F^-^ intake and low incidence of CKD–CTN despite high F^-^ intake [8, 9]. For example, personal observations and published data exemplify that water F^-^ contents as low as 0.5 mg/L (i.e., acceptable limits) cause dental fluorosis in children in some villages in dry zones. Ironically, chronic water ingestion with F^-^ concentration above 3 mg/L in other regions and America does not cause dental or skeletal flurosis [8, 9].

**Fig. 4:**
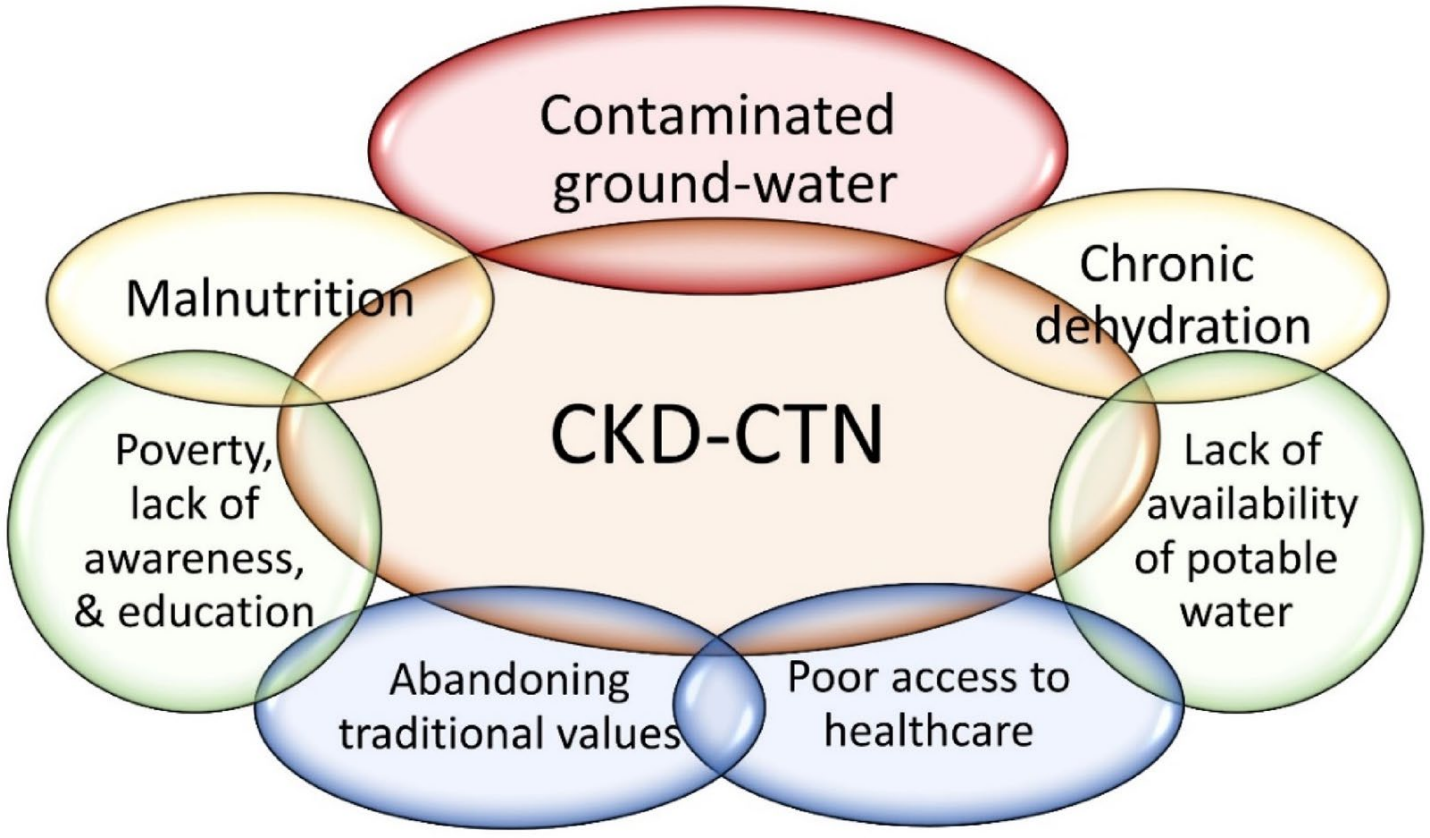
Key factors that contribute to the development of CKD–CTN. These are necessary prerequisites to create an internal milieu in renal tissues to precipitate mineral crystals and nano-tubes. The estimated proportions of individual contributions are illustrated in the degree of overlaps

In vitro and in vivo experiments designed with bioavailability components and varied ratios (e.g., Ca^2+^/Mg^2+^), in clinically relevant dose exposures, compared to controls, are essential to make sensible conclusions. In humans, it takes over 10 years of exposure to develop CKD–CTN. Therefore, future research should simulate realistic environmental conditions, such as concentrations, temperature, duration of exposure, and appropriate physical activity in age-specific animal models relevant to humans [53]. Such experiments are valuable for understanding the in vivo conditions causing nano-crystal formation and the additional ways to mitigate them [82, 83]. Prospective, multi-discipline, multifaceted, and multi-centre clinical studies using realistic dose/duration exposure relevant to humans are warranted [10]. Figure 4 summarises multiple factors that work together to provide a conducive micro-environment and increase the vulnerability of persons to contract CKD–CTN.

### Reasons why CKD–CTN has not been controlled to date

Despite that mentioned evidence, the CKD–CTN-affected communities are neglected. Governments in none of the affected countries have made it a priority to control or eradicate this deadly disease. All these countries are classified as developing economies and failed to prioritise preventing this CRF. Affected communities are located in rural regions, and peasants live in a subsidiary economy with daily labour. They do not have a voice to get the attention of the government. Despite these, most of the work of provision of clean water conducted today is by individual philanthropists and smaller charitable organisations.

Larger charities have not been involved in helping the affected villages, as there is not much for them to gain from these rural communities. What has successfully reduced the incidence of CKD–CTN in the affected regions is providing potable water, preventing dehydration, and changing lifestyles. Construction of renal clinical, dialysis units, and even renal hospitals had no impact on reducing the incidence of CKD–CTN, as they all work on the tail-end of this fatal disease. Overall, respective governments have taken only a slight interest in providing clean water, which too was done haphazardly in a few locations. There is a widespread conflict of interest among politicians, bureaucrats, and those who have made this fatal disease a significant business, including intermediaries, governmental departments, and doctors.

## Conclusion

Longer term consumption (exposure) of stagnant groundwater concentrated with ions and chronic dehydration creates a favourable internal milieu for CaPO_4_ and other mineral crystallisation in kidneys. Over-exploitation of groundwater and prolonged droughts lead to increased poor-quality groundwater and lack of potable water, aggravated by unhealthy behavioural issues that collectively cause this crystal-tubular nephropathy (CTN). Because of the described Geo–Bio pathways (Fig. 2) leading to disease, the populations that live in the immediate geo-environment are the most vulnerable to developing CKD–CTN [10].

Consumption of stagnant groundwater with high ion content over many years with chronic dehydration due to lesser water intake is necessary for crystal development in vivo. The ions and minerals in those stagnant wells get concentrated due to annual prolonged dry periods. This makes such groundwater unpalatable; therefore, residents consume less water. Daily alcohol intake worsens chronic dehydration, creating a perfect storm in the internal milieu in renal tissues—a conducive in vivo environment for crystal formation.

Willful destruction of the environment through greed-driven, lass-scale deforestation, over-extraction of groundwater for agriculture, irresponsible behaviour of people that endanger nature, poorly planned human settlements, and disregarding the environmental consequences have paved the path for the genesis of this deadly kidney disease. These frequent fires and flooding, large-scale waste- air- and water pollution, and prolonged droughts threaten human and animal health, including chronic renal failure—CKD–CTN.

The mechanisms that cause CKD–CTN are illustrated here to facilitate an understanding of the actions needed to overcome CKD–CTN. It also provides cost-effective early intervention strategies to prevent and eradicate CKD–CTN from tropical countries. Besides the nano-crystal/nano-tube concept (Fig. 3), there is overwhelming evidence that a balanced diet enriched with micronutrients, antioxidants (selenium, Zn, etc.), and Mg^2+^ protect kidneys from all forms of CKDs, especially the crystal formation of CKD–CTN.

Providing potable water, increasing water consumption, and avoiding harmful behaviour are fundamental in affected regions to protect the renal health of farm labourers and others who regularly engage in strenuous physical work in hot and dry environments. CKD–CTN is a chronic, Geo–Bio mediated disease that arises from natural causes (unrelated to agrochemicals or heavy metals), epitomised by multiple environmental and behavioural factors. With the proactive deployment of strategic practices and economic measures, CKD–CTN can be entirely preventable and eradicable. 

Historical evidence shows that ancient Kingdoms in Sri Lanka of Anuradhapura, Polonnaruwa, Dambadeniys, Sigiriya, Yapahuwa, etc., time to time had been moved to new locations, mainly within the dry zonal regions that are currently affected by CKD-CTN. Whether these forced relocations were made for better protect from invading armies, severe malarial epidemics, or deaths due to CKD-CTN during those periods, is uncertain: perhaps the combination of all three.

## Summary

CKD–CTN is caused by the consumption of concentrated and naturally contaminated groundwater (i.e., underground, geological reasons), where people live for decades: this fulfils the Bradford–Hill criteria of causation. The described novel concepts facilitate a deeper understanding of the aetiology of CKD–CTN and provide cost-effective opportunities to prevent the development of renal failure and premature deaths. A fundamental understanding of the new concept of developing crystalline nephropathy, causing CKD–CTN, paves the path for cost-effective targeted solutions to protect peasants. In addition to providing potable water for affected communities, multiple interventions are necessary to overcome this silent killer [52]. Further research will confirm these concepts and develop improved, practical solutions.

Such approaches would save thousands of lives of people in CKD–CTN-affected tropical countries who regularly engage in strenuous physical work in hot environments and consume insufficient water. It is crucial to systematically address lifestyles and dietary habits to protect their renal health and improve micro-nutrition to mitigate CKD–CTN in vulnerable populations. The way to eradicate CKD–CTN is by preventing the disease, not by aggressively treating end-stage renal diseases, such as expanding renal clinics, dialysis centres, and renal transplantation services.

## Data Availability

Not applicable.
